# A correlative microscopy approach relates microtubule behaviour, local organ geometry, and cell growth at the *Arabidopsis* shoot apical meristem

**DOI:** 10.1093/jxb/ert352

**Published:** 2013-10-23

**Authors:** Agata Burian, Michał Ludynia, Magalie Uyttewaal, Jan Traas, Arezki Boudaoud, Olivier Hamant, Dorota Kwiatkowska

**Affiliations:** ^1^Department of Biophysics and Morphogenesis of Plants, University of Silesia, Jagiellońska 28, 40-032 Katowice, Poland; ^2^Laboratoire de Reproduction et Développement des Plantes, INRA, CNRS, ENS, UCB Lyon 1, France; ^3^Laboratoire Joliot Curie, CNRS, ENS Lyon, Université de Lyon, 46 Allée d’Italie, 69364 Lyon Cedex 07, France

**Keywords:** *Arabidopsis thaliana*, cortical microtubules, growth, mechanical stress, organ geometry, shoot apical meristem.

## Abstract

Cortical microtubules (CMTs) are often aligned in a particular direction in individual cells or even in groups of cells and play a central role in the definition of growth anisotropy. How the CMTs themselves are aligned is not well known, but two hypotheses have been proposed. According to the first hypothesis, CMTs align perpendicular to the maximal growth direction, and, according to the second, CMTs align parallel to the maximal stress direction. Since both hypotheses were formulated on the basis of mainly qualitative assessments, the link between CMT organization, organ geometry, and cell growth is revisited using a quantitative approach. For this purpose, CMT orientation, local curvature, and growth parameters for each cell were measured in the growing shoot apical meristem (SAM) of *Arabidopsis thaliana*. Using this approach, it has been shown that stable CMTs tend to be perpendicular to the direction of maximal growth in cells at the SAM periphery, but parallel in the cells at the boundary domain. When examining the local curvature of the SAM surface, no strict correlation between curvature and CMT arrangement was found, which implies that SAM geometry, and presumed geometry-derived stress distribution, is not sufficient to prescribe the CMT orientation. However, a better match between stress and CMTs was found when mechanical stress derived from differential growth was also considered.

## Introduction

Morphogenesis relies on the ability of gene networks to regulate mechanical properties and growth of individual cells. In turn, changes in the mechanical properties, growth, and organ geometry feed back on the regulatory networks, and this is commonly believed to increase the robustness of morphogenesis (Besnard *et al.*, 2011). However, the existence of such feedbacks renders their analysis difficult, as, for example, growth becomes both a cause and a consequence of morphogenesis.

Plant cells are joined together by the continuous system of rigid cell walls, and a given shape can be generated only by the irreversible deformation (plastic strain) of walls, which is at the basis of growth. Growth is usually anisotropic [i.e. it attains different values in different directions (Dumais and Kwiatkowska, 2002; Coen *et al.*, 2004; Baskin, 2005)]. The main cause of growth anisotropy has been related to the anisotropic texture of cell walls, which comprise a network of stiff cellulose microfibrils. Although this suffers some exceptions, the spatial organization of microfibrils is generally thought to be guided by cortical microtubules (CMTs) (Green, 1962; Giddings and Staehelin, 1991; Lloyd, 2011). CMTs target the delivery of vesicles containing cellulose synthase to the plasma membrane (Crowell *et al.*, 2009; Gutierrez *et al.*, 2009) and guide the trajectory of cellulose synthase complexes at the membrane (Paredez *et al.*, 2006).

Consistent with the role of CMTs in guiding cellulose deposition, CMT orientation is usually perpendicular to the direction of maximal growth in fast growing cells (Baskin *et al.*, 1999; Sugimoto *et al.*, 2000). Cell growth also generates mechanical stress, and both stress and strain (reversible and irreversible) may in turn affect CMT orientation. It has been proposed that CMTs respond to growth (strain) by orienting perpendicular to the direction of maximal growth (Fischer and Schopfer, 1997, 1998). However, data from the literature also provide contradictory results. Transverse orientation of CMTs can precede a phase of rapid elongation (Chan *et al.*, 2011), suggesting that growth may not be the initial trigger for CMT orientation. Furthermore, auxin induces CMT reorientation from longitudinal into transverse in non-elongating cells, showing that growth is not necessary for CMT reorganization (Takesue and Shibaoka, 1999). It has therefore been proposed that cells are able to detect growth directions and, via a positive feedback mechanism, subsequently reinforce the direction of maximal growth by orienting CMTs perpendicular to this direction (Fischer and Schopfer, 1998). In this framework, growth is both a consequence and a cause of a particular CMT orientation, which poses one of the difficulties in studying the relationship between growth and CMTs. As the impact of CMTs on cell growth is indirect, another difficulty comes from a potential delay in the ‘translation’ of CMT orientation into cellulose microfibril organization, and a ‘readout’ of cell wall structure into anisotropy of cell growth.

It has also been proposed that CMTs orient according to mechanical stress directions in tissues. Different experimental approaches show that the CMTs orient along the predicted direction of maximal stress in cell walls (Hejnowicz *et al.*, 2000; Hamant *et al.*, 2008). By orienting the cellulose microfibrils along the maximal stress direction, CMTs would create a negative feedback loop, causing cells to resist the forces that are exerted on them. This was investigated recently at the shoot apical meristem (SAM) of *Arabidopsis thaliana* by Hamant *et al.* (2008). The SAM, a group of continuously growing and dividing cells, is a dome-shaped structure with primordia appearing first as bulges at its periphery (see, for example, the review by Kwiatkowska, 2008). Between the primordium and the apical dome, a saddle-shaped boundary forms, which consequently becomes a sharp crease that separates the growing primordium from the SAM. Hamant *et al.* (2008) compared qualitatively the predicted distribution of mechanical stress with the CMTs. Assuming the SAM surface is relatively stiff and subjected to pressure from internal tissues, the SAM is analogous to a pressure vessel having the same shape (Selker *et al.*, 1992; Dumais and Steele, 2000; Hamant *et al.*, 2008). In a continuous model based on this assumption, the distribution of mechanical stress can be predicted from the SAM geometry at the global (tissue) scale: the stress is isotropic in the spherical apical dome, and anisotropic, with the maximum in the circumferential (latitudinal) direction, at the cylindrical SAM flanks (Hamant *et al.*, 2008). In the saddle-shaped boundary, the predicted stress is tensile in the latitudinal direction and compressive in the meridional. The predicted stress directions corresponded, at least qualitatively, to the global CMT organization (Hamant *et al.*, 2008). Since the predicted direction of maximal stress globally corresponds to the direction of maximal curvature, a premise from this model is that CMT orientation is parallel to the direction of maximal curvature. This is, however, not systematically the case, and even neighbouring cells in the spherical dome can have different CMT orientations (Uyttewaal *et al.*, 2012). This can be explained by assuming that local differences in cell growth rate and/or changes in mechanical properties of cell walls can generate forces that are independent of the local curvature (e.g. Selker *et al.*, 1992).

Recently, it has also been demonstrated that overall cell geometry can determine CMT orientation by affecting the CMT polymerization (Ambrose *et al.*, 2011): CMTs encountering a sharp edge (e.g. an edge between a newly formed anticlinal cell wall and a periclinal wall) are more likely to depolymerize. Accordingly, CMT orientation parallel to sharp edges is favoured. However, the effect of edge sharpness can be mitigated by the microtubule-associated protein CLASP, which provides a local stabilization of CMTs.

Although in principle they are different, the stress- and strain-based hypotheses can lead to the same overall behaviour of CMTs. For example, in elongating cylindrical cells of *Nitella*, the CMTs (transverse to the cell axis) are parallel to the predicted direction of maximal stress (transverse) and perpendicular to the direction of maximal growth (Gertel and Green, 1977; Wasteneys and Williamson, 1987). However, in the basal region of sunflower hypocotyl (still elongating), both the maximal stress and the maximal growth direction in the outer epidermal cell wall are longitudinal (parallel to the organ axis). In this case, the CMTs tend to be parallel to the direction of maximal stress and maximal growth (Hejnowicz *et al.*, 2000).

Observations of elongating organs as well as of the SAM (Hamant et al, 2008; Uyttewaal *et al.*, 2012) would rather argue for the stress-based hypothesis. It should be noted, however, that both stress- and strain-based hypotheses are supported mostly by qualitative assessments of SAM geometry, growth, and CMT orientation. Therefore, the link between CMT behaviour, local SAM geometry, and cell growth was revisited using a quantitative and correlative microscopy approach with cellular resolution. In this context, the SAM represents an ideal system to study, as directions of growth vary in space and change in time during initiation and development of new organs in a predictive manner. In addition, the complexity of the local geometry changes provides a stringent and rigorous context for such an analysis.

## Materials and methods

### Plant material and growth conditions

The GFP–MBD (green fluorescent protein–microtubule-binding domain) line of *A. thaliana* was kindly provided by Martine Pastuglia (INRA, Institut Jean-Pierre Bourgin, France). Plants were grown first in short-day conditions (8h light/16h dark period at an illumination of 100 μmol m^–2^ s^–1^) for 2 or 3 weeks, and next in long-day conditions (16h/8h), at a temperature of 22 °C. Shoot apices were cut from inflorescences (3–9cm long), all flower buds that covered the SAM were removed, and such dissected apices were transferred to Apex Culture Medium (Supplementary Materials and methods available at *JXB* online). Dissected apices in the medium were kept in a plant growth chamber (MLR-351H, Panasonic) in long-day conditions (16h light/8h dark period at 100 μmol m^–2^ s^–1^) at 22 °C.

### Sequential imaging by confocal laser scanning microscopy

To visualize CMTs in the SAM outermost layer (L1), a confocal laser scanning microscope was used (Zeiss LSM 510) equipped with a long working distance water immersion objective (Achroplan 40×/0.8W), and the laser emitting at a wavelength of 488nm. Stacks of sections taken at 1 μm and 0.5 μm intervals in the Z direction (for short-term and long-term kinetics, respectively), 1.4–2× zoom, and frame averaging 4, were collected at 30–35% of laser power. The process of scanning of each SAM took ~5–10min. In the case of short-term observation, the images were acquired at nine time points with 20min intervals; in the case of long-term observation, they were taken at two or three time points with 24h intervals. The first observation in the sequence was performed 3–11h after the apex dissection. Between consecutive observations, apices were kept in the growth chamber.

### Sequential replica method and imaging by scanning electron microscopy

To obtain data necessary for computation of curvature and growth variables, the sequential replica method was used as described previously (Dumais and Kwiatkowska, 2002). Briefly, impressions of the individual SAM surface were taken using the silicon dental impression material (Take 1, Kerr impression materials), no later than 2h after the SAM imaging in the confocal microscope. The impressions were filled with epoxy resin (Devcon 2 ton epoxy). Casts obtained in this way were sputter-coated and imaged by scanning electron microscopy (Philips XL 30 TMP ESEN). For each cast, a stereopair of images was taken to enable three-dimensional (3D) reconstruction of the SAM surface.

### Analysis of CMT alignment

Stacks of confocal images were first processed in MerryProj software (Barbier de Reuille et al., 2005) to obtain the 2D projection of CMTs located under the outer periclinal cell walls of the SAM L1 layer. To quantify the mean orientation of CMTs and the anisotropy of the CMT array in individual cells, ImageJ was employed (National Institutes of Health; downloaded from http://rsbweb.nih.gov/ij/) with a macro developed to measure the intensity of the fluorescent signal (Supplementary Fig. S1 at *JXB* online; Uyttewaal *et al.*, 2012). Such computed values of the CMT anisotropy range from 0 when the orientation of CMTs in a cell is fully random, to 1 when the CMTs are aligned all in the same direction. For each analysed image, the anisotropy was normalized according to the formula: *x*_norm_=*x*_i_/*x*_max_, where *x*_norm_ is a normalized anisotropy value; *x*_i_ is an original value; and *x*_max_ is the maximal value for the image. Since the CMT orientation is a directional variable, in order to quantify local variability of CMT orientation and the mean CMT orientation in groups of cells, statistics for circular data were employed (Zar, 1999; Berens, 2009), using circular standard deviation (SD) as a measure of local variability. Groups of cells used for this computation comprised a given cell and its five closest neighbours. CMT orientation or the direction of maximal growth/curvature were described referring to the general shape of the SAM that can be approximated by a sphere, and the terms meridional and latitudinal, which are equivalent to radial and circumferential in 2D projection, were used. In polar plots, the following terms were used to describe CMT orientation or maximal growth direction with respect to the meridional SAM direction: meridional for 0–30 ° or 150–180 °; latitudinal for 60–120 °.

### Computation of local SAM geometry and cell growth

All codes used for the quantitative analysis of geometry and growth were written in Matlab (The Mathworks, Natick, MA, USA). Descriptive statistics, statistical tests, and plots have been performed with the aid of Matlab Statistics Toolbox and Origin (OriginLab Corporation, USA).

Quantification of SAM geometry and growth, including the 3D reconstruction of the surface, segmentation into cells, computation of curvature, and growth parameters, was performed with the aid of previously described protocols (Dumais and Kwiatkowska, 2002; Routier-Kierzkowska and Kwiatkowska, 2008; see also Supplementary Materials and methods at *JXB* online).

### Merging data on CMTs and growth/curvature parameters

To integrate data obtained from confocal microscopy and scanning electron microscopy imaging, two transformation matrices were computed using original Matlab protocols. The first matrix (*T*_1_) mapped the 3D data obtained from the surface reconstruction over the 2D confocal projection data; and the second one (*T*_2_) mapped the 2D data over the 3D data (Supplementary Materials and methods at *JXB* online). The transformations were represented by the 4×4 matrices accounting for translation, rotations in *XY*, *YZ*, and *XZ* planes, and scaling (such transformation matrices are described in detail in Barbier de Reuille et al., 2005).

## Results

To relate CMT orientation to local organ geometry and cell growth during morphogenesis at the SAM of *Arabidopsis*, a correlative microscopy approach was developed, integrating quantitative data coming from live imaging with the aid of confocal microscopy and the sequential replica technique coupled with scanning electron microscopy (Supplementary Fig. S2 at *JXB* online). Briefly, using the dissected shoot apex of a GFP–MBD-expressing line, the GFP signal from the outermost SAM layer (L1) was numerically extracted to observe the CMT arrays under the outer periclinal cell walls (Barbier de Reuille et al., 2005). For the same apex, the SAM surface was reconstructed in 3D and segmented into cells, based on replica images from scanning electron microscopy (Routier-Kierzkowska and Kwiatkowska, 2008). To quantify CMT orientation and anisotropy of the CMT array in each cell, an earlier developed tool was used (Uyttewaal *et al.*, 2012), while protocols designed for the replica technique allowed quantification of local curvature and growth in the same cells (Dumais and Kwiatkowska, 2002; Routier-Kierzkowska and Kwiatkowska, 2008). To integrate the quantitative data on CMTs, curvature, and growth, original protocols were developed (see the Materials and methods).

### Adjacent cells with similar CMT orientation tend to have more stable CMT alignments

**Fig. 1.**
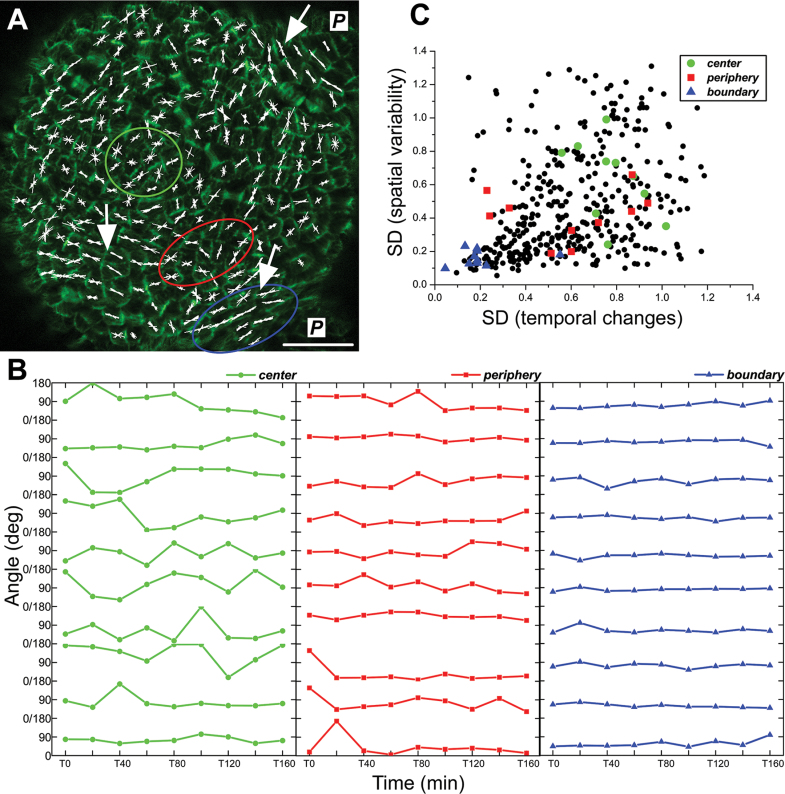
Short-term kinetics of CMT reorientation. (A) CMT projection at T=0min. Line segments (white) represents the mean CMT orientation in a cell; their length is proportional to the anisotropy of the CMT array. Segments in each cell are from T=0min to T=160min (measurements at 20min time interval). Domains at the SAM centre, periphery, and a boundary between the apical dome and a primordium (P) at which the analysis of CMT kinetics (B) was performed are outlined. Arrows indicate good examples of groups of cells, where CMTs are stable and well aligned across adjacent cells. Bar=20 μm. (B) CMT orientation in individual cells at T=0min to T=160min measured with respect to the horizontal axis of the image. Ten cells from domains outlined in (A) are shown: the SAM centre (green), the periphery (red), and the boundary (blue). (C) Correlation between spatial variability and temporal changes of CMT orientations. Spatial variability is represented as a circular SD (weighted by CMT anisotropy) of CMT orientation computed at T0min for a cell and its five closest neighbours. Temporal changes are measured as the SD (weighted by CMT anisotropy) of CMT orientation computed for individual cells from T=0min to T=160min. Data from two SAMs are shown (*n*_total_=409 cells, including *n*_SAM1_=213 and *n*_SAM2_=196 cells). Values for cells from different domains are marked in green, red, and blue, and for the remaining cells in black. Spearman’s rank-order correlation coefficient is 0.43 (statistically significant correlation at *P*=0.05).

Since CMTs are dynamic and can undergo continuous reorientation within minutes or hours in both hypocotyls and meristems (Hejnowicz, 2005; Chan *et al.*, 2007; Hamant *et al.*, 2008), the short-term kinetics of CMT reorientation at the SAM were first checked, as a reference for the long-term (24h time interval) observations. For this purpose, CMTs were imaged every 20min for 160min and their orientation and anisotropy were quantified in individual cells at each time point (Fig. 1A).

Similar to what was found in a previous qualitative study (Hamant *et al.*, 2008), CMTs reorient more in the SAM centre than in the periphery or boundary (Fig. 1B). To quantify these temporal changes in CMT orientation (CMT reorientation), the circular SD of CMT orientation in individual cells during the entire duration of measurements was computed (Supplementary Fig. S3A at *JXB* online). Changes of CMT orientation were highly correlated with specific SAM domains. The SD was ~2.5 times higher in the centre than in the boundary. In addition, the mean anisotropy of CMT arrays (i.e. the degree of CMT alignment within a given cell) was 45% lower in the centre than in the boundary (Supplementary Fig. S3B). In other words, in the centre, CMTs were randomly oriented and changed their orientation more frequently than in the boundary. In the SAM periphery, a mosaic of both reorienting and stable CMT arrays was observed, exhibiting various degrees of anisotropy (Fig. 1B; Supplementary Fig. S3B). More specifically, while in the boundary and in the centre neighbouring cells exhibited similar CMT temporal change and degrees of anisotropy, the periphery was much less uniform, with clusters of cells with stable and similar CMT orientation (Fig. 1A, arrows) surrounded by cells with less stable orientation. Interestingly, lower local variability of CMT orientation among adjacent cells (lower spatial variability) correlated with relatively stable CMTs (i.e. maintaining a roughly constant orientation in time) (Fig. 1C; Spearman’s rank-order correlation coefficient=0.43). In contrast, a high local variability of CMTs between adjacent cells was correlated with continuous CMT reorientation.

The important premise from this analysis is that on the basis of the distribution of CMT orientation in space, one can reach conclusions on their change in time. In the following, CMT behaviour, growth, and curvature parameters were analysed based on long-term kinetics.

### There is no strict and uniform correlation between CMT orientation and maximal growth direction throughout the SAM

**Fig. 2.**
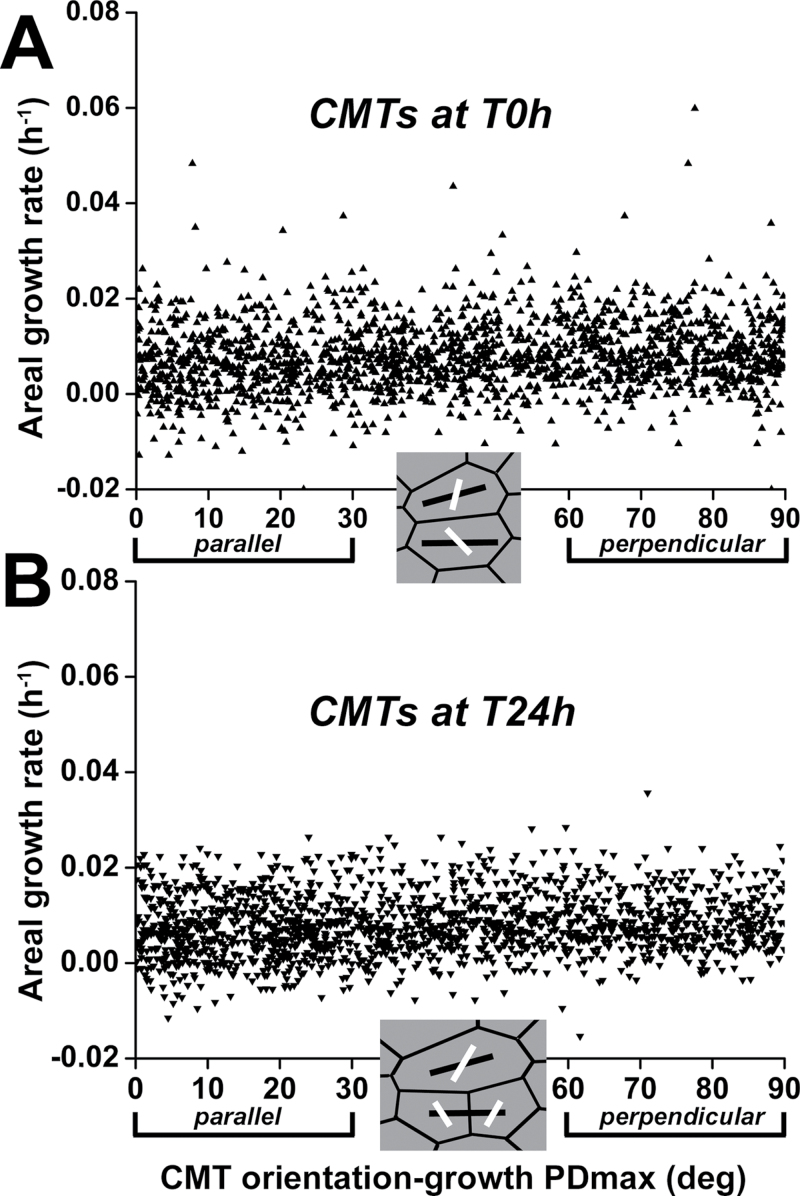
Difference between CMT orientation and direction of the maximal growth rate (growth PDmax) plotted against areal growth rate in individual cells. Growth rates were computed for the 24h time interval. (A) and (B) refer to CMT orientation at the first and the second time points (at the beginning and at the end of the interval), respectively. Data for cells from five SAMs replotted (*n*=1822 in A, *n*=1726 cells in B). Insets show good examples of cells with maximal growth direction (black line segments) and CMT orientation (white) at the first (A) and second (B) time points.

Next, the link between CMT orientation and the direction of maximal growth rate was investigated. Recently, in hypocotyl epidermis, CMT orientation has been shown to be highly variable at the outer periclinal face, while it is strictly transverse to the direction of maximal growth at the inner face (Crowell *et al.*, 2011; Fujita *et al.*, 2011). To check if this is also the case at the SAM, CMT orientation at both the outer and inner faces of the L1 layer was quantified. In the majority (83%) of cells examined (*n*=297 cells from five SAMs), the difference between mean CMT orientation at the outer and inner faces was <30 ° (Supplementary Results 1,  Supplementary Fig. S4, Supplementary Movie 1 at *JXB* online). Thus, in contrast to hypocotyl cells, CMTs at the SAM L1 layer exhibit a similar orientation at both the outer and inner cell faces. Accordingly, growth rates in principal directions (directions of either maximal or minimal growth rate) were computed for the outer periclinal cell walls for 24h time intervals, and CMT orientations under these walls were quantified both at the first and at the second time points (at the beginning and at the end of the time interval, respectively). The analysis was based on the following assumptions: (i) CMTs are regarded as stable if the orientation is the same at the beginning and the end of the time interval; (ii) CMT orientation determines cellulose microfibril organization with a small delay; and (iii) the maximal growth direction is transverse to the recently deposited microfibrils. To check whether CMTs are parallel or perpendicular to the maximal growth direction, the difference between the maximal growth direction and the CMT orientation was computed for individual cells. Pooling all the cells from five SAMs, this difference was plotted against areal growth rate (Fig. 2).

In the case of the CMTs at the first time point, CMTs were oriented perpendicular, oblique, or parallel to the direction of maximal growth independently of growth rate (Fig. 2A). The same was true for CMTs at the second time point (Fig. 2B).

All these quantifications demonstrated that the SAM as a whole exhibits no uniform correlation between CMT orientation and maximal growth direction. Since the SAM comprises domains differing in timing of CMT reorientation and morphogenetic events, the relationship between CMTs and growth was next analysed in further detail in the central zone (CZ), peripheral zone (PZ), and boundary.

### Stable CMTs are perpendicular to the maximal growth direction in the peripheral zone but parallel in the boundary

**Fig. 3.**
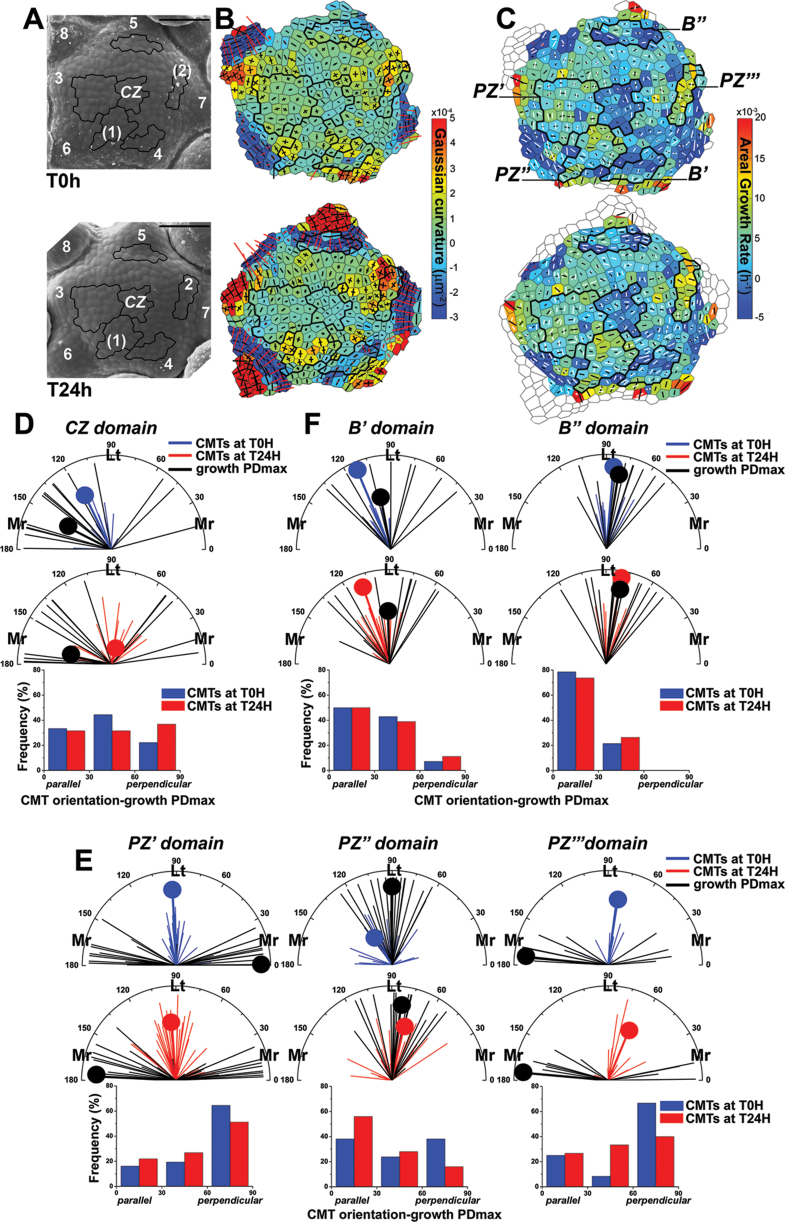
Relationship between CMT orientation and maximal growth direction for cells in the selected SAM domains. (A) Scanning electron micrograph of the representative SAM at the first (T0h, upper panel) and the second (T24h, lower panel) time points. Primordia are numbered starting from the youngest (numbers of incipient primordia are in parentheses). The selected domains are outlined in black. Bar=50 μm. (B) Corresponding curvature maps at the first and the second time points. Gaussian curvature is represented in the colour scale; and curvatures in principal directions as cross arms (for positive values in black, for negative in red). (C) Growth rate maps and overlaid CMTs. The areal growth rate is represented in the colour scale; the maximal growth direction (growth PDmax) is given as a black line segment, and the mean CMT as a white line segment. These parameters were plotted onto the cell outlines at the first (T0h, upper panel) and the second (T24h, lower panel) time points. (D–F) Polar plots of CMT orientation and the direction of growth PDmax for the first (T0h, upper panels) and the second (T24h, middle panels) time points at the CZ (D), PZ (E), and boundary (F) domains. Thin line segments represent CMT orientation (blue for T0h, red for T24h) or growth PDmax (black) in individual cells; the segment length is proportional to CMT or growth anisotropy. Thick line segments (tipped with a dot) represent the circular mean CMT orientation or mean growth PDmax (weighted by the anisotropy); its length is the measure of a dispersion of data: the longer the segment, the more data are concentrated around the mean orientation/direction. Mr refers to meridional CMT orientation or growth PDmax (0–30 °; 150–180 ° with respect to meridional SAM direction); and Lt to latitudinal orientation (60–120 °). Histograms of the difference between CMT orientation and the growth PDmax for the first (T0h, blue bars) and for the second (T24h, red bars) time points (lower panels) at the CZ (D), PZ (E), and boundary (F) domains. CMT orientation is regarded as parallel to the growth PDmax for the difference 0–30 °, and perpendicular for 60–90 °. Cell numbers are: *n*_CZ_=18 (T0h) and 19 (T24h); *n*_PZ′_=31 (T0h) and 41 (T24h), *n*_PZ′′_=21 (T0h) and 25 (T24h), n_PZ′′′_=9 (T0h) and 12 (T24h); *n*_B′_=14 (T0h) and 18 (T24h), and *n*_B′′_=14 (T0h) and 19 (T24h).

For each cell, the CMT orientation and maximal growth direction were determined with respect to the meridional direction (the radius) of the SAM dome, as well as the difference between CMT orientation and the direction of maximal growth (Fig. 3). Domains at the CZ, PZ, and boundaries were selected based on growth, and local geometry described by the Gaussian curvature (a product of curvatures in the principal directions, i.e. the maximal and minimal curvatures). The Gaussian curvature indicates different morphogenetic events at the SAM (Kwiatkowska, 2008). For example, a relatively high Gaussian curvature indicates the bulge—a future primordium, whereas the negative curvature is usually restricted to a saddle-shaped crease that forms at the boundary between the primordium and the SAM. Five SAMs were analysed, and in all the cases similar relationships between CMT orientation and the direction of maximal growth were noted, as shown for the representative SAMs (Fig. 3; for another example, see Supplementary Results 2, Supplementary Fig. S5 at JXB online).

First the CZ domain was analysed, defined by its central position in the SAM and a relatively low areal growth rate (Fig. 3A–C). Gaussian curvature was low but positive in this domain. The dispersion of both CMT orientation and maximal growth direction among cells was relatively high (Fig. 3D). CMTs were randomly oriented with respect to the growth direction, which may be related to the fact that CMTs were especially dynamic in the CZ, as revealed by short-term kinetics of CMTs (Fig. 1). Therefore, while a correlation might exist between the growth direction and CMT orientation in the CZ, the highly dynamic CMTs in this domain hindered any conclusion.

Next, domains located at the PZ were analysed (Fig. 3E). As growth at the PZ is generally not uniform, this zone was subdivided into three subdomains (PZ′, PZ′′, and PZ′′′), in which the maximal growth direction of adjacent cells was locally uniform (Fig. 3A–C). PZ′, located between a bulging primordium (primordium 3 in Fig. 3A, B) and CZ, is a domain where no organogenesis is taking place, Gaussian curvature is low but positive, and most cells exhibit maximal growth in the meridional direction (Fig. 3C, E) and relatively stable latitudinal CMT arrays. Clearly then, CMTs in this domain are perpendicular to the maximal growth direction. PZ′′ and PZ′′′ include cells contributing to the initial bulge, as marked by an increase in Gaussian curvature at the second time point (Fig. 3A, B). The initial bulge is regarded as a rudimentary bract (Kwiatkowska, 2006; Alvarez-Buylla *et al.*, 2010). Its formation is the earliest stage of flower primordium development in *Arabidopsis* and precedes the formation of the flower primordium proper. The PZ′′ represents the earlier stage of the initial bulge formation, while PZ′′′ represents the later stage. In the PZ′′ domain, the maximal growth was almost uniformly in the latitudinal direction, but the dispersion of CMT orientation was relatively high (ranging from latitudinal to meridional) and the mean CMT orientation changed between two time points (Fig. 3C, E). Thus, during this time interval, CMTs could be temporarily parallel, oblique, or perpendicular to the maximal growth direction in this domain. In the PZ′′′ domain, the maximal growth was in the meridional direction, and CMTs tended to be latitudinal; that is, perpendicular to the direction of maximal growth (Fig. 3C, E).

Finally, CMT arrays in boundary domains of earlier (B′) and later (B′′) stages were analysed (Fig. 3F). In the boundary domain B′′ with an apparent crease, where the Gaussian curvature is negative (Fig. 3A–C), both CMT orientation and direction of maximal growth were latitudinal or nearly latitudinal (Fig. 3F). In other words, CMTs were parallel to the maximal growth direction. A similar tendency was also observed in a younger (i.e. shallower), boundary B′.

In summary, this analysis further rules out growth direction as an instructive signal for CMT orientation, as CMTs can be either parallel or perpendicular to the maximal growth direction, depending on the morphogenetic events at the domain. This analysis also reveals that as cells change their position from the SAM centre to the forming organ, trends in the relationship between CMTs and growth direction are changing. The CMT orientation can be random with respect to maximal growth direction in the CZ. In PZ′ they become perpendicular to this direction, while in PZ′′ CMTs can be unstable and temporarily parallel, oblique, or perpendicular to the maximal growth direction, and again perpendicular in PZ′′′. Finally, in the boundary domain, CMTs are stable and parallel to the maximal growth direction.

Next it was tested to what extent the CMTs are quantitatively related to the local SAM geometry, in order to investigate whether the local predicted stress derived from geometry (following a pressure vessel analogy) is sufficient to guide CMT behaviour in meristematic cells.

### CMT orientation is not strictly correlated to local SAM geometry

**Fig. 4.**
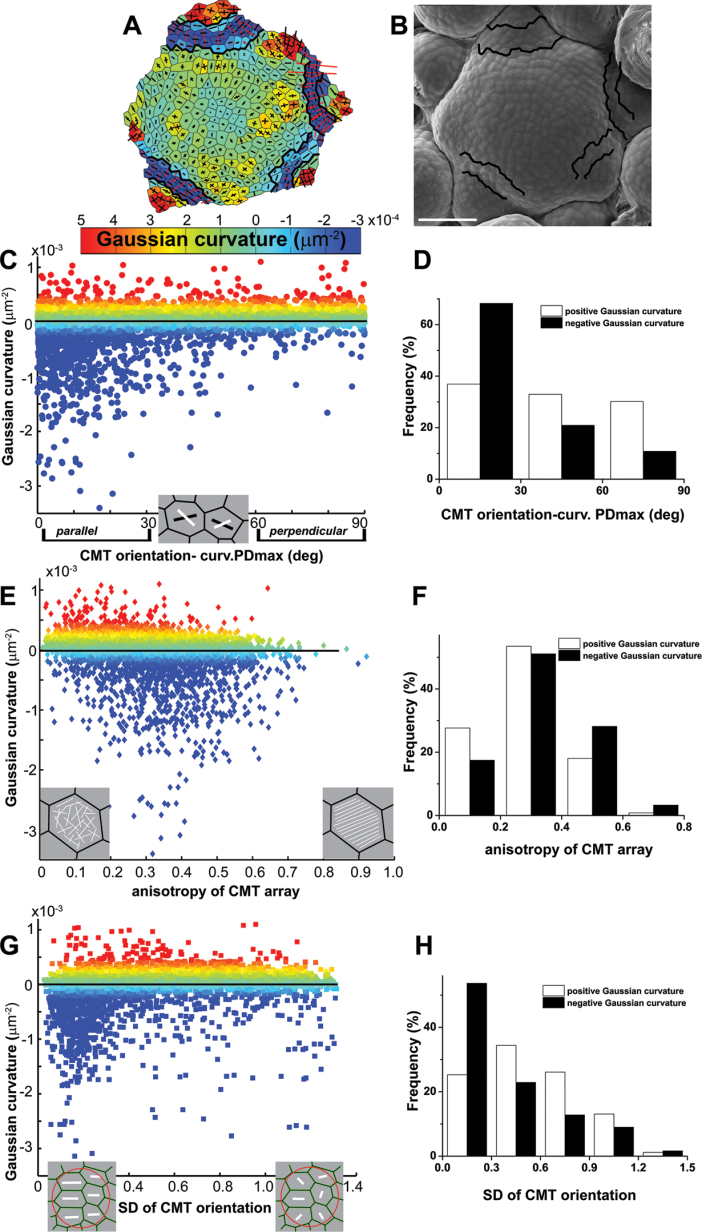
Relationship between CMT organization and local SAM geometry in individual cells. (A and B) Curvature map (A) and corresponding scanning electron micrograph (B) for a good example of a SAM. Boundaries between the SAM and primordia are outlined. Bar=50 μm. (C) Difference between CMT orientation and the direction of maximal curvature (curvature PDmax) plotted against Gaussian curvature. The colour scale (Gaussian curvature) is the same as for the curvature map in (A). The inset shows good examples of cells with curvature PDmax (black line segment) and CMT orientation (white). (D) A histogram of the difference between CMT orientation and curvature PDmax with either positive (white bars) or negative (black bars) Gaussian curvature. (E) Anisotropy of CMT arrays plotted against Gaussian curvature. The Spearman’s rank-order correlation coefficient equals –0.21 (statistically significant at *P*=0.05). The inset shows a good example of a cell with a CMT array of low anisotropy (disordered CMTs) and with a CMT array of high anisotropy (ordered CMTs). (F) A histogram of anisotropy of CMT arrays in individual cells with either positive (white bars) or negative (black bars) Gaussian curvature. (G) Local variability of CMT orientation plotted against Gaussian curvature. Local variability is assessed by a circular SD of CMT orientation computed for a cell and its five closest neighbours. The inset shows a good example of a group of six cells with CMTs (white line segment) of lower and higher variability for which the circular SD was computed. (H) A histogram of local variability of CMT orientation (SD) with either positive (white bars) or negative (black bars) Gaussian curvature. In (C–H) cells from eight SAMs are plotted (*n*=6415 cells).

Assuming that the SAM surface is much stiffer than the inner tissues, the distribution of stresses can at least partially be deduced from the SAM geometry (Dumais and Steele, 2000). In particular, the predicted direction of maximal stress mostly corresponds to the direction of maximal curvature (Hamant *et al.*, 2008). As mentioned earlier, it has been proposed that CMT orientation follows the direction of maximal stress in cell walls. To extend the previous analysis to more local variations in the SAM curvature, experiments were carried out to test whether at the local (cellular) scale there is a relationship between CMTs and curvature (Fig. 4).

First the difference between CMT orientation and the direction of maximal curvature was computed. Pooling all the cells from eight SAMs, this difference was plotted against Gaussian curvature (to associate the difference with the position at the SAM, Gaussian curvature was also plotted in the colour scale, the same as in the curvature maps) (Fig. 4A–C). Interestingly, the difference between CMT orientation and the direction of maximal curvature was related to the sign of Gaussian curvature. If the Gaussian curvature was positive, there was no constant, prevailing CMT orientation with respect to the direction of maximal curvature (Fig. 4D). Locally, CMTs could be oriented in small groups of cells in the latitudinal direction as described earlier (Hamant *et al.*, 2008); however, taken together, 37% of CMT arrays exhibited parallel, 33% oblique, and 30% perpendicular orientation with respect to the direction of maximal curvature. However, if the curvature was negative as in the boundary domains, the majority of cells exhibited CMT orientation parallel to the maximal curvature direction (68% parallel, 21% oblique, and 11% perpendicular orientation).

Secondly, it was checked whether CMT anisotropy in individual cells is correlated with Gaussian curvature, and a weak correlation was found (Spearman’s rank-order correlation coefficient= –0.21): the lower the curvature, the higher the CMT anisotropy (Fig. 4E, F).

Thirdly, it was tested if local curvature can orchestrate the behaviour of CMTs among neighbouring cells. For this purpose, it has been investigated whether supracellular patterns of CMTs, distinguished by a low variability of CMT orientation between adjacent cells, are related to a specific surface geometry. A circular SD of CMT orientation (see the Materials and methods) assigned for a given cell and its five closest neighbours was plotted against the Gaussian curvature assigned for this cell (Fig. 4G). Although only a weak correlation was found (Spearman’s rank-order correlation coefficient=0.27), the formation of supracellular CMT patterns was increased if the Gaussian curvature was negative: 54% of the cell groups exhibited low CMT variability (SD ≤0.3) if the curvature was negative, whereas this was only 25% in the case of positive curvature (Fig. 4H).

To summarize, by examining the SAM curvature at the local level, it was shown that at SAM domains of positive Gaussian curvature there is no strict correlation between CMT organization and the curvature direction. However, it was confirmed that at domains of negative curvature, such as boundaries, CMTs more frequently formed supracellular patterns and were mostly parallel to the maximal curvature direction, thus a predicted maximal stress direction.

**Fig. 5.**
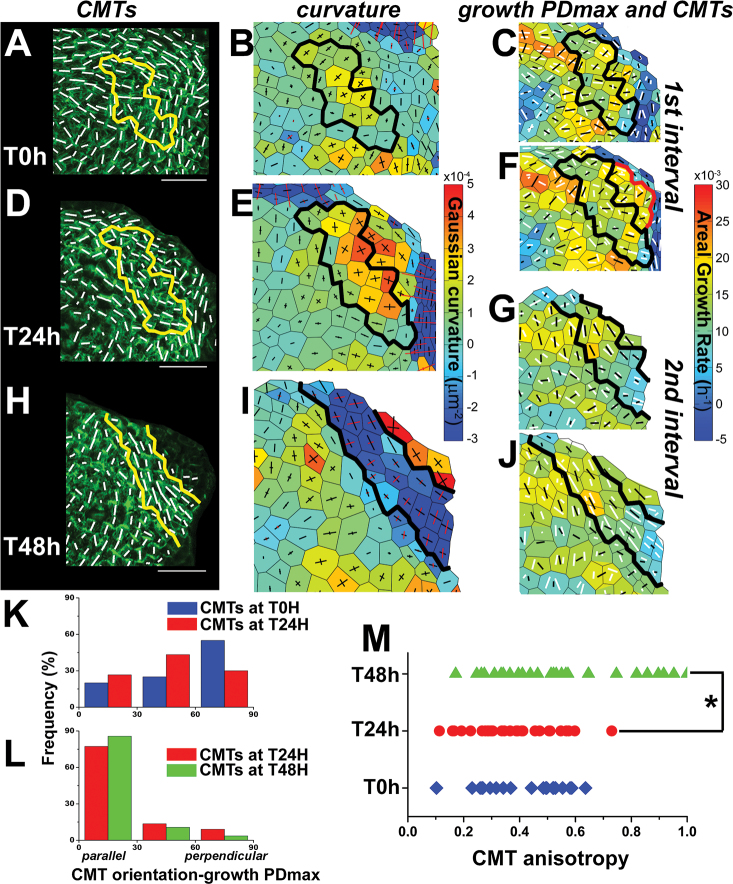
Changes of CMT orientation, curvature, and maximal growth directions during boundary formation. The boundary domain (outlined) was identified at the T48h point (H and I) and the same domain was recognized at T0h and T24h. (A, D, and H) CMT projections at the first (T0h) (A), the second (T24h) (D), and the third (T48h) (H) time points. White segments represent the mean CMT orientation in a cell. Bar=20 μm. (B, E, and I) Corresponding curvature maps at the first (T0h) (B), the second (T24h) (E), and the third (T48h) (I) time points. (C, F, G, and J) Corresponding growth rate maps with overlaid CMTs. Growth at the boundary domain (outlined) is shown for two 24h time intervals: the first interval from T0h to T24h (C and F), and the second from T24h to T48h (G and J). Growth PDmax is represented as a black segment, and CMT orientation at a given time point as a white segment. Growth parameters in the first interval are plotted onto the cell outlines as they appeared in the first (T0h) (C) and the second (T24h) (F) time points; those for the second interval are plotted onto the cell outlines at the second (T24h) (G) and the third (T48h) (J) time points. The mean areal growth rate of cells at the emerging bulge (r_bulge_ in F, outlined in red) and of cells at the future boundary (r_boundary_ in F, outlined in black) is r_bulge_=0.0175 (SD=0.0022; SE=0.0009) and r_boundary_=0.0137 (SD=0.0037; SE=0.0010); the difference between means is statistically significant (*t*-test at *P*=0.05). (K and L) Histograms of the difference between CMT orientation and growth PDmax in individual cells at the boundary domain for the first (K) and the second (L) time intervals (blue bars for CMTs at T0h, red at T24h, and green at T48h) (*n*=20, 30, and 28 cells at T0h, T24h, and T48h). (M) Anisotropy of CMT arrays in individual cells at the boundary domain at T0h (blue), T24h (red), and T48h (green). An asterisk indicates a significant difference between the distributions (Kolmogorov–Smirnov test at *P*=0.05).

Next, changes in CMT organization were analysed during the formation of boundaries that are presumptive sites of highly anisotropic stress, with the maximum (tension) in the latitudinal direction and the minimum (compression) in the meridional direction. As the boundary folds (deepens), the anisotropy of stress is expected to increase and to affect CMT orientation and behaviour. To test this prediction and relate CMTs to the evolution of the boundary shape over time, CMT orientation was followed simultaneously with growth and local curvature during formation of the boundary at three consecutive time points (Fig. 5).

A representative case is detailed: at the first time point (T0h), the curvature at the future boundary domain was positive, and CMTs did not form any supracellular pattern (Fig. 5A, B, outlined). CMTs were oriented mainly perpendicular to the maximal growth direction (Fig. 5C, K). At the second point (T24h), the Gaussian curvature remained positive and was increased in comparison with T0h (Fig. 5E), which indicates that the outlined domain, together with adjacent cells, was at the stage of initial bulging. However, despite the fact that the crease was not formed yet, there was an apparent CMT arc-like pattern of nearly latitudinal orientation (Fig. 5D). CMTs at this point were oblique with respect to the maximal growth direction in the preceding time interval (Fig. 5F, K). At the third time point (T48h), Gaussian curvature was negative in the domain and the arc-like CMT pattern seemed to be more pronounced (Fig. 5H, I). Clearly, CMTs became stable and parallel to the maximal growth direction (Fig. 5G, J, L). In addition, the process of boundary formation was associated with an increase in the anisotropy of CMT arrays in individual cells (Fig. 5M; see also another example in Supplementary Fig. S6 at *JXB* online).

Altogether, the quantitative analysis presented in this study implies that local SAM geometry, and associated predicted stress distribution, are not sufficient to determine the CMT orientation. Nevertheless, assuming that cells respond to geometry-derived stresses above a certain threshold of stress anisotropy, a correlation with stress may be drawn as the best correlation between SAM geometry and CMT behaviour was found as the crease in the boundary becomes sharper. Furthermore, geometry is not the sole source of stress: stress may also arise from differential growth between adjacent domains of the SAM. In particular, CMT reorganization at the future boundary occurs in a region where the growth rates can be locally very different. Indeed, as the emerging primordium is growing faster than the boundary domain (at the first time interval the mean areal growth rate of cells in a bulge is ~1.3 times higher than that of cells in the future boundary), the differential growth possibly generates a latitudinal stress around the emerging organ before the tissue folds, and this stress may be sufficient to orient and further stabilize the CMTs. Thus, it is proposed that mechanical stress, derived from both differential growth and SAM geometry, is the best predictor of CMT behaviour in the SAM.

## Discussion

The correlative microscopy approach presented in this study relates CMT behaviour, as quantified on confocal image projections with a tensor-based tool (Uyttewaal *et al.*, 2012), to the cell growth rate and local SAM geometry quantified for scanning electron microscopy images obtained with the replica method (Dumais and Kwiatkowska, 2002; Routier-Kierzkowska and Kwiatkowska, 2008). These two types of data were integrated using original protocols. Applying such a quantitative strategy, three main results were obtained. First, temporal changes of CMTs in a group of adjacent meristematic cells can be predicted from the CMT organization in these cells. Secondly, while CMTs are classically thought to be perpendicular to the direction of maximal growth in plant cells, at least in the boundary domain of the SAM CMTs were instead parallel to the direction of maximal growth. Thirdly, as it was shown that CMTs were not always oriented parallel to the direction of maximal curvature, it is concluded that organ geometry *per se*, and associated geometry-derived stress distribution, is not sufficient to determine the CMT orientation at a local level. However, during boundary formation, CMT arrangement matched with the predicted maximal stress direction when considering stress derived from both local geometry and differential growth.

### Temporal changes in CMT orientation

CMTs are extremely dynamic. Their reorientation can be induced by various extrinsic factors, such as light, hormones, or mechanical forces (Williamson, 1991; Shibaoka, 1994; Hamant *et al.*, 2008; Sambade *et al.*, 2012; Vineyard *et al.*, 2013). CMTs can also undergo an intrinsic constant reorientation (Mayumi and Shibaoka, 1996; Takesue and Shibaoka, 1998; Hejnowicz, 2005; Chan *et al.*, 2007). This intrinsic reorientation could account for the CMT reorientation revealed by short-term kinetics in the CZ and PZ domains of the SAM.

Linking short-term kinetics of CMT reorientation with CMT spatial organization in the present study, it was found that there is a correlation between temporal changes of CMT orientation in a cell and local variability of CMT arrays between adjacent cells. Namely, cells of relatively stable and well-aligned CMTs were usually clustered in groups with similar CMT orientation, thus forming a supracellular pattern. It has been reported that although CMTs undergo constant non-synchronous reorientation in adjacent cells of the hypocotyl epidermis, particular CMT orientations (transverse, longitudinal, or oblique) can be stable over several hours and well aligned across cells (Chan *et al.*, 2011). This observation is consistent with the existence of a supracellular, rather than cell-based, factor. In view of previous studies (Cyr, 1994; Williamson, 1990, 1991; Landrein and Hamant, 2013) and the present study, a mechanical signal is proposed to contribute to this supracellular organization.

### Relationship between CMTs and growth

Although recent articles highlight the role of mechanical stress in orienting CMTs in the SAM, the idea that CMTs respond to growth is still debated, notably because a positive feedback loop between microtubules and growth could provide a simple way to maintain microtubule orientations in growing organs (Fischer and Schopfer, 1998). Using a quantitative approach in the present study, whether a response to growth could explain the CMT behaviour in the SAM was tested.

When pooling all cells from the examined CMTs and computing the difference between CMT orientation and maximal growth direction, no strong trends were found. On the one hand, cases were found (e.g. in certain domains of the PZ) where CMTs were perpendicular to the maximal growth rate, consistent with the earlier proposed positive feedback loop (Fischer and Schopfer, 1998; Fisher and Cyr, 1998); an increased growth rate in the meridional direction would induce CMT latitudinal orientation, which by affecting the deposition of cellulose microfibrils in cell walls would amplify growth in the meridional direction. This meridional growth accounts for some stages of initial bulging, and ‘rebuilding’ of PZ in domains, where no organogenesis takes place (Kwiatkowska, 2006). On the other hand, this scenario does not apply to other SAM domains. In particular, at the boundary domain, CMTs were strictly parallel to the maximal growth direction. Since CMTs (and cellulose microfibrils) can also be aligned in the maximal growth direction in other plant model systems such as *Vinca major* shoot apex or *Graptopetalum paraguayense* residual meristem (Jesuthasan and Green, 1989; Green and Selker, 1991) this seems to be a more universal phenomenon that disproves the hypothesis that CMTs are always oriented perpendicularly to the maximal growth.

Due to the technical limitations on the time lapse between two observations, all the above conclusions are based on the simplest assumptions about the link between CMTs and growth. Therefore, in domains with less stable CMTs, more complex behaviours stemming from, for instance, delays between CMTs and cell wall structure, or between the resulting microfibril organization throughout the whole cell wall and growth direction, cannot be excluded.

### Relationship between CMTs and mechanical stress derived from organ geometry

The alternative hypothesis that CMTs orient parallel to the direction of maximal stress has been tested before (Hejnowicz *et al.*, 2000; Hamant *et al.*, 2008). However, the main difficulty in its verification is that, in contrast to the measurable growth, there is no technique for direct stress measurement in plant shoot apices. Thus, a description of stress is only speculative, predicted from indirect observations or from theoretical considerations (Hussey, 1971, 1973; Lynch and Lintilhac, 1997; Dumais and Steele, 2000; Hamant *et al.*, 2008; Moulia *et al.*, 2011; Uyttewaal *et al.*, 2012). According to a continuous model based on the assumption that the SAM surface is stiffer than internal tissues, the supracellular stress distribution can be deduced from the global geometry of the SAM (Hamant *et al.*, 2008). In particular, the maximal stress direction mostly corresponds to the direction of maximal curvature. While this model globally fitted with the CMT behaviour in the different regions of the SAM (Hamant *et al.*, 2008), it was not tested at the local (cellular) scale. In particular, one would predict that the local geometry of the SAM could lead a local stress pattern that would impact on CMT orientation.

In this study, a quantitative approach was used to correlate the CMT orientation in individual cells to the maximal curvature direction. When pooling all cells from the examined SAMs, CMTs were not always oriented parallel to the curvature direction. More specifically, it was found that the relationship between CMTs and the curvature direction depends on the sign of Gaussian curvature: if the Gaussian curvature was positive, there was no prevailing CMT orientation with respect to maximal curvature direction, whereas CMTs were oriented parallel to the curvature direction if the Gaussian curvature was negative, as in the boundary domains. This is consistent with the prediction that such negative Gaussian curvature should dramatically increase the anisotropy of mechanical stress, and thus provide a clear directional signal to orchestrate CMT behaviour among adjacent cells. However, quantifications demonstrated that the arc-like supracellular CMT pattern is formed before negative curvature appears, thus indicating that geometry-derived stress is not sufficient to prescribe the CMT behaviour at the SAM.

The relationship between CMT organization and geometry could in fact be more complex due to the action of microtubule-associated proteins. For instance, it has been proposed that, by default, CMTs orient parallel to sharp cell edges, unless they are stabilized by CLASP (Ambrose *et al.*, 2011). While the sharpness of edges might be correlated with local organ curvature, data in the present study do not allow this issue to be addressed.

### Relationship between CMTs and mechanical stress derived from differential growth

Stress in plant cell walls results from several factors acting at both cellular and supracellular scales. Accordingly, the stress resulting from turgor pressure can be modified due to different mechanical properties of cells from different SAM domains (Milani *et al.*, 2011; Peaucelle *et al.*, 2011; Kierzkowski *et al.*, 2012), changes in the cell wall properties induced by auxin or expansins (Burgert and Fratzl, 2006), differences in the mechanical properties of cell layers (Hejnowicz and Sievers, 1996), or growth rates of adjacent cells or tissues (Peters and Tomos, 1996; Vandiver and Goriely, 2008). For example, compressive stress may build up in the rapidly growing region of the meristem surface delimited by the slow-growing regions (Selker *et al.*, 1992; Green *et al.*, 1996). Consistent with previous studies (Kwiatkowska, 2006; Uyttewaal *et al.*, 2012), in the present study it was noted that the initial bulging stage, which precedes boundary formation, is associated with a strong growth gradient. Namely, the areal growth rate locally increases in cells, forming a bulge, resulting in a growth gradient with a maximum at the bulge. Such differences in growth rates (differential growth) may generate stress, in addition to the stress derived from the SAM geometry. Accordingly, a theoretical model predicts that a primordium outgrowth leads to a circumferential (latitudinal) stress around the primordium (Hamant *et al.*, 2008). Therefore, it is proposed that the differential growth during initial bulging generates a latitudinal stress before tissue folds, and that this stress is sufficient to orient the CMTs along the future boundary. In this scenario, mechanical stress would determine the CMT behaviour in a two-step process at both the cellular and supracellular scale: first, differential growth between the bulging primordium and the future boundary induces a latitudinal stress that stabilizes the CMTs in the latitudinal orientation across adjacent cells. Secondly, as the tissue is folding, the emergent geometry amplifies the latitudinal stress, thus further stabilizing the CMTs and increasing CMT alignment in the cells.

### Specific case of the boundary

Assuming that CMT orientation closely corresponds to the orientation of cellulose microfibrils at the SAM (Hardham *et al.*, 1980), it is concluded that in the boundary, cells grow more in the direction of cellulose reinforcement generated by CMTs. This unexpected observation would be one of the unusual cases where a structural cell wall anisotropy determined by the reinforcement is overcome by additional factors. Because it is extremely anisotropic in that domain, mechanical stress could be one of these factors (Baskin, 2005). Similarly, cells at the flanks of the *V. major* shoot apex grow in the direction of the reinforcement when subjected to a rapid directional stretch caused by the stress generated in adjacent primordia (Jesuthasan and Green, 1989). Growth in the direction of cellulose microfibrils was also described in elongating stems or roots (Paolillo, 2000; Wiedemeier *et al.*, 2002). It has been postulated that the growth direction might also depend on properties of microfibrils, or interactions between microfibrils and other wall components (Wasteneys, 2004; Baskin, 2005; Fujita *et al.*, 2011). It is also possible that cell walls in the boundary have specific mechanical properties.

## Conclusions

Whereas the results presented in the present study are in line with the concept that CMT behaviour is governed by mechanical factors, it remains to be established how this could work. Stress could be sensed very locally at the level of the cell wall, the cytoskeleton, or the plasma membrane. It is possible that cells sense stress directly by a resulting elastic strain. However, in growing cells, plastic strain (growth) accompanies the elastic strain. The method applied here for growth rate measurements does not allow discrimination between elastic and plastic strain. CMT behaviour is probably also closely coupled to other factors, such as hormone gradients, diverse cell wall modifications, or the presence of proteins that influence cytoskeleton dynamics (Lloyd and Hussey, 2001; Pien at al., 2001; Reinhardt *et al.*, 2003; Heisler *et al.*, 2005; Peaucelle *et al.*, 2008, 2011, Ambrose *et al.*, 2013). Unravelling interactions between these processes will require an integrative approach involving quantitative analysis as described herein.

## Supplementary data

Supplementary data are available at *JXB* online.

Supplementary Materials and methods.

Figure S1. Quantification of CMT organization using ImageJ macro.

Figure S2. Scheme of the protocol for the integration of data from the replica method and CMT live imaging.

Figure S3. Short-term kinetics of CMT reorientation. Analysis of standard deviation and CMT anisotropy.

Supplementary Results 1 and Figure S4. CMT organization at the outer and inner periclinal faces of the SAM L1 layer.

Supplementary Results 2 and Figure S5. Relationship between CMT orientation and maximal growth direction for cells in the selected SAM domains.

Figure S6. Changes of CMT orientation and anisotropy of CMT array during boundary formation.

Supplementary Movie 1. CMT organization across the SAM layers.

Supplementary Data

## Abbreviations:

CMT: cortical microtubule
curvature/growth PDmax: the direction of maximal curvature/growth
CZ: central zone
GFP: green fluorescent protein
MBD: microtubule-binding domain
PZ: peripheral zone
SAM: shoot apical meristem.
