# Supplementary material for: A correlative microscopy approach relates microtubule behaviour, local organ geometry, and cell growth at the Arabidopsis shoot apical meristem
Source: J Exp Bot. 2013 Oct 23;64(18):5753–67. doi: 10.1093/jxb/ert352 (PMC3871827; doi:10.1093/jxb/ert352)
Supplement: Supplementary Data [file supp_64_18_5753__index.html]

A correlative microscopy approach relates microtubule behaviour, local organ geometry, and cell growth at the Arabidopsis shoot apical meristem — A correlative microscopy approach relates microtubule behaviour, local organ geometry, and cell growth at the Arabidopsis shoot apical meristem — Supplementary Data 

# A correlative microscopy approach relates microtubule behaviour, local organ geometry, and cell growth at the *Arabidopsis* shoot apical meristem

## Supplementary Data

Data files

**Files in this Data Supplement:**

- Supplementary Data - Supplementary Data
- Supplementary Data - Supplementary Data
