# Supplementary material for: A correlative microscopy approach relates microtubule behaviour, local organ geometry, and cell growth at the Arabidopsis shoot apical meristem
Source: J Exp Bot. 2013 Oct 23;64(18):5753–67. doi: 10.1093/jxb/ert352 (PMC3871827; doi:10.1093/jxb/ert352)
Supplement: Supplementary Data [file supp_ert352_jexbot103010_file001.pdf]

Journal of Experimental Botany

A correlative microscopy approach relates microtubule behavior, local organ geometry, and cell growth at the Arabidopsis shoot apical meristem

Agata Burian, Michał Ludynia, Magalie Uyttewaal, Jan Traas, Arezki Boudaoud,
Olivier Hamant, Dorota Kwiatkowska

Supplementary Data

Contents:

Supplementary Materials and Methods
Supplementary Figure S1
Supplementary Figure S2
Supplementary Figure S3
Supplementary Results 1, Figure S4, Movie S1
Supplementary Results 2, Figure S5
Supplementary Figure S6
Supplementary References

Supplementary Materials and Methods

Plant Material and Growth Condition

Dissected shoot apices were grown *in vitro* in ACM medium (Apex Culture Medium: 0.5x Murashige and Skoog minimal organic powder medium, 1 % sucrose, 0.8 % agarose, vitamins: 1 μl/1 ml of 1000x stock, 50 ml consisting of 5 g Myo-inositol, 0.05 g Nicotinic acid, 0.05 g Pyridokine hydrochloride (Vitamin B6), 0.5 g Thiamine hydrochloride (Vitamin B1), 0.1 g Glycine, pH= 5.8).

Computation of Local SAM Geometry and Cell Growth

To quantify of SAM geometry and growth, the SAM surface was first reconstructed and 3D coordinates of vertices (points where three cells are in the contact) were computed. Coordinates of vertices of a given cell and all its contacting neighbors were used to compute principal directions of curvature, and minimal and maximal curvatures. Gaussian curvature was computed as a product of minimal and maximal curvatures. To quantify growth parameters, the same vertices were recognized at the consecutive replica images based on clonal analysis. Principal directions of growth rates at vertices were computed on the basis of linear transformation mapping positions of three natural vertex neighbors (the vertices connected with a given vertex by common anticlinal cell walls) before growth onto their positions after growth. Growth rates in principal directions (minimal and maximal growth rates, $growth_{min}$ and $growth_{max,}$ respectively) for a cell were computed as a mean of the growth rates of its vertices. Growth anisotropy of a cell was computed as abs($growth_{min}$ - $growth_{max}$) / (abs($growth_{min}$) + abs($growth_{max}$)). The anisotropy values range between 0 and 1, for isotropy and maximal anisotropy, respectively. Areal growth rate of a cell was computed as ($growth_{min}$ + $growth_{max}$).

Merging Data on CMTs and Growth/Curvature Parameters

In order to compute the T_1, first a set of vertices from the 3D reconstructed surface was recognized at the corresponding 2D confocal projection of CMTs. Since the GFP-MBD signal was present at each cell facet the cell contours were visible at the stack sections, which facilitated recognition of vertex positions at the confocal projection. Second, based on the corresponding sets of 3D and 2D vertex coordinates the transformation matrix was computed with the aid of Solver (add-in for Microsoft Office Excel), so that the distance between corresponding vertices was minimized. Generally, depending on a quality of replicas and

confocal stacks, the identification of 50-200 vertices per SAM (i.e. 9-30 cells from different regions of the SAM) was enough to provide satisfactory results. The T_1 matrix was next applied to transform all vertices and cell center coordinates from the reconstructed 3D surface onto the 2D CMT projection. As a result, non-directional parameters of geometry or growth of individual cells (Gaussian curvature and areal growth rate) were assigned to the corresponding CMT parameters.

Next, matrix T_2 was computed as the inverse of T_1. This matrix was used to integrate directional parameters, i.e. CMT orientation and principal directions of curvature or growth. For this purpose first the 2D data on CMT orientation (i.e. line segments defining the mean CMT orientation in a cell) were transformed with the aid of T_2, and second each line segment representing CMT orientation was projected on the plane defined by the crosses representing principal directions. Then angles between CMT orientation and principal directions of curvature or growth rate in individual cells were computed for each cell.

Usually the number of cells that can be recognized at the replica images was lower than that recognized at the confocal stacks. Unlike in the confocal stacks, in the replica images new anticlinal cell walls were visible with a delay, because the groove in the replica surface, which is formed above the new anticlinal wall, is initially too shallow. Thus, in replicas the youngest sister cells appeared as a single cell. In such cases, two (13-30% of all cases), or occasionally three (< 2%) or four (< 1%), values of CMT parameters were regarded as corresponding to the same geometry or growth parameters attributed to one cell at the reconstructed surface.

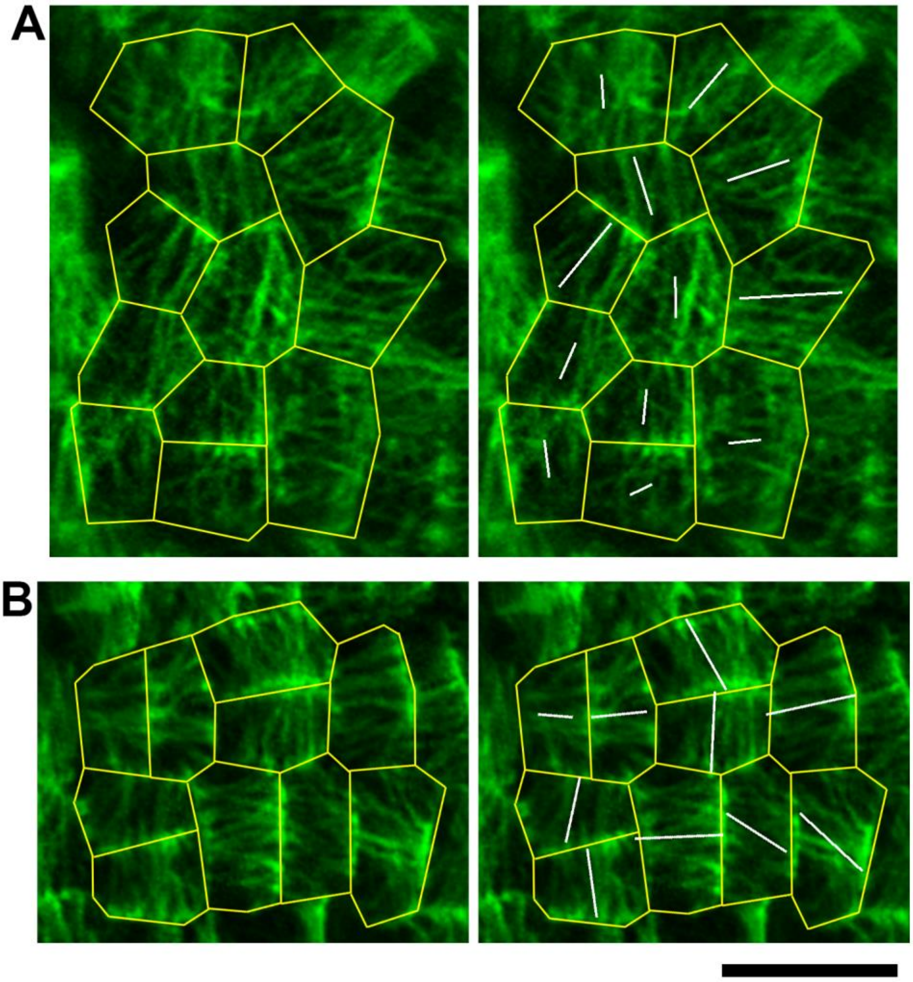

Supplementary Figure S1. Quantification of CMT organization using ImageJ macro. CMT projections from the outer periclinal cell face of L1 layer, (A) SAM center, (B) SAM periphery. Cells are outlined in yellow with (right panel) or without (left panel) overlaid output from ImageJ. Mean CMT orientation in a cell and anisotropy of the array are visualized as a white line segment, which orientation represents the mean CMT orientation and length is proportional to the anisotropy. Bar = 10 µm.

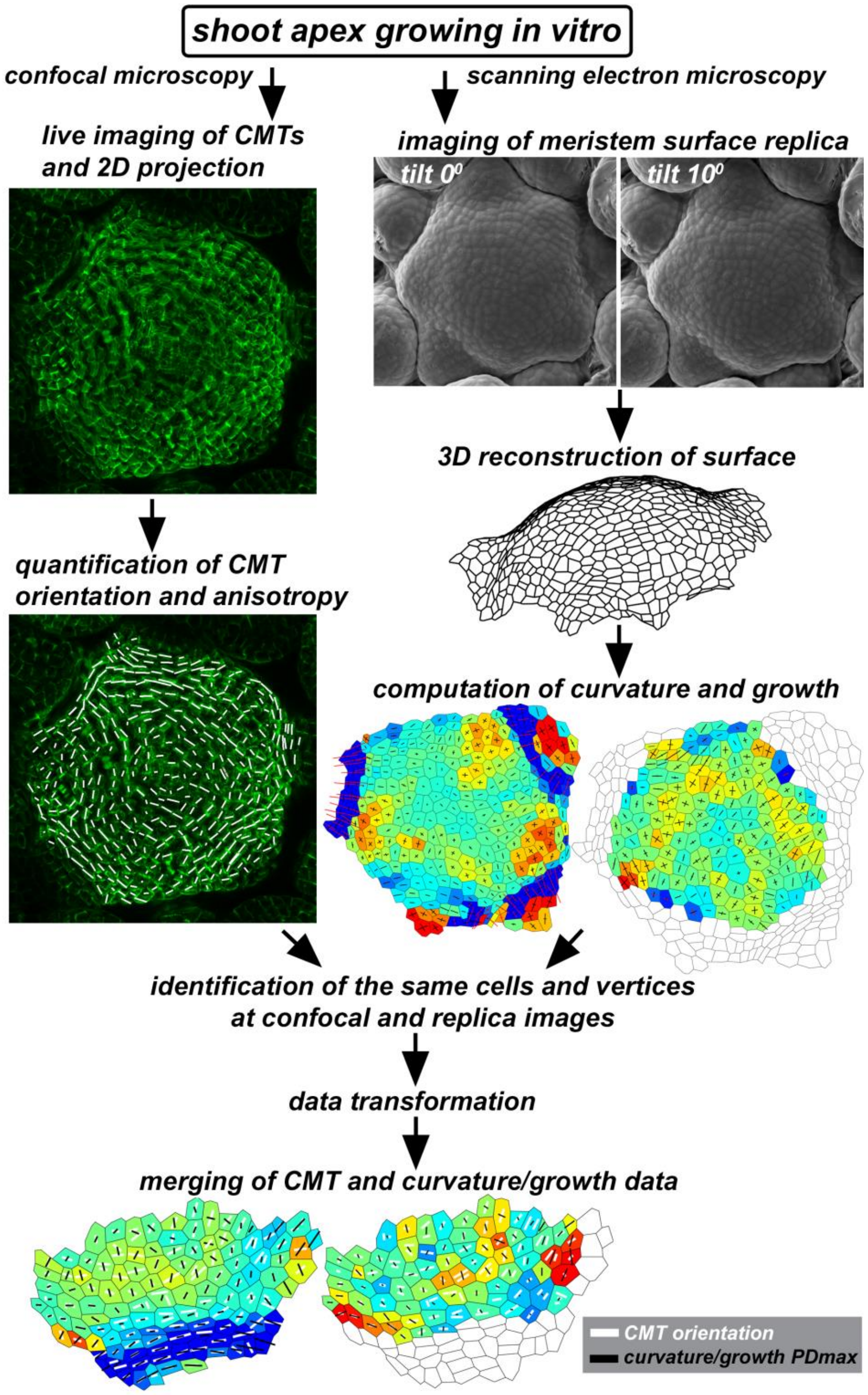

Supplementary Figure S2. Scheme of the protocol for the integration of data from replica method and CMT live imaging. PDmax refer to directions of maximal curvature or growth.

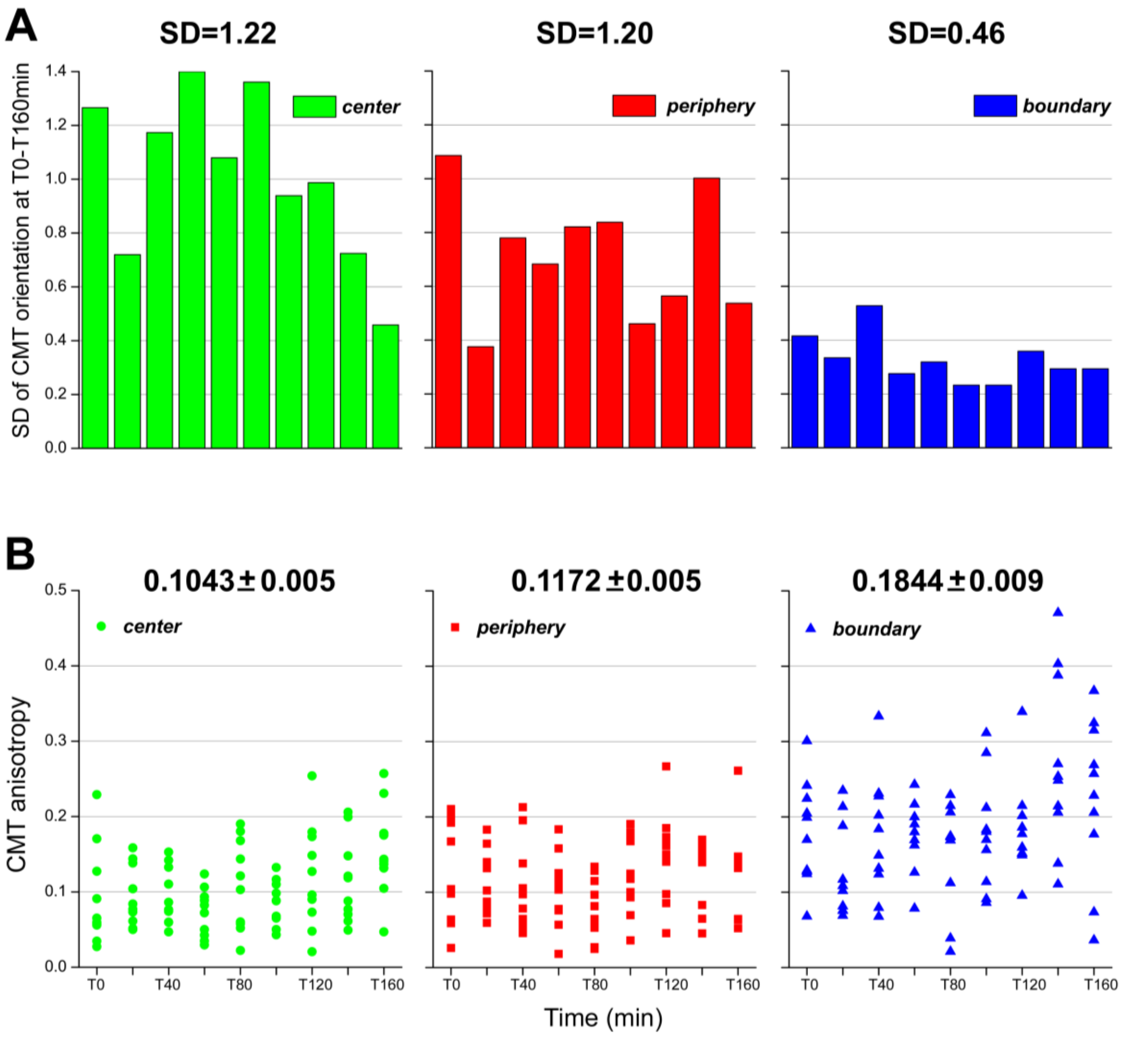

Supplementary Figure S3. Short-term kinetics of CMT reorientation. Analysis of a standard deviation (SD) and CMT anisotropy. (A) A circular SD of CMT orientation from T=0 min to T=160 min for ten individual cells from the SAM domains outlined in Fig. 1A: the SAM center (green bars), periphery (red), and the boundary (blue). Numbers at the top of each plot indicate the circular SD computed for all cells pooled from a particular domain. (B) Anisotropy of CMT array at T=0 min to T=160 min for ten cells from the SAM domains outlined in (Fig. 1A): the SAM center (green dots), periphery (red dots), and boundary (blue dots). The mean anisotropy ± standard error (SE) is given for each domain.

Supplementary Results 1. CMT organization at the outer and inner periclinal faces of L1 layer.

Given that the CMT organization differs between the inner and the outer faces of the hypocotyl epidermis (Crowell *et al.,* 2011; Fujita *et al.,* 2011), this issue was addressed also in the SAM. CMTs at the outer and inner face of the same cells were extracted from the confocal stacks, and then the mean CMT orientation and anisotropy were quantified in each cell face. Generally, CMT orientation was similar at outer and inner faces. In particular, at the SAM center CMTs were rather disordered at both faces, whereas CMTs were ordered at SAM periphery and boundaries on both faces (Supplementary Fig. S4, A-D). Accordingly, when quantifying the mean CMT orientation at a given face, the difference between outer and inner face in 83% cells was not higher than 30^0 (Supplementary Fig. S4E).

To check if the difference depends on CMT anisotropy (the lower anisotropy, the higher difference), the difference was plotted against the anisotropy of CMTs either at outer or inner face, but no strong tendency was found (Supplementary Fig. S4, F and G). However, the anisotropy of CMT arrays at inner face was slightly lower than that at outer face (the mean anisotropy for outer and inner face are 0.12 and 0.08, respectively). In other words, whereas CMTs could be well aligned at the outer face, they could be more disordered at the inner face (Supplementary Fig. S4, A and B, yellow arrows). Lower anisotropy might partially result from a higher noise in the CMT measurements, due to difficulties in imaging the inner face.

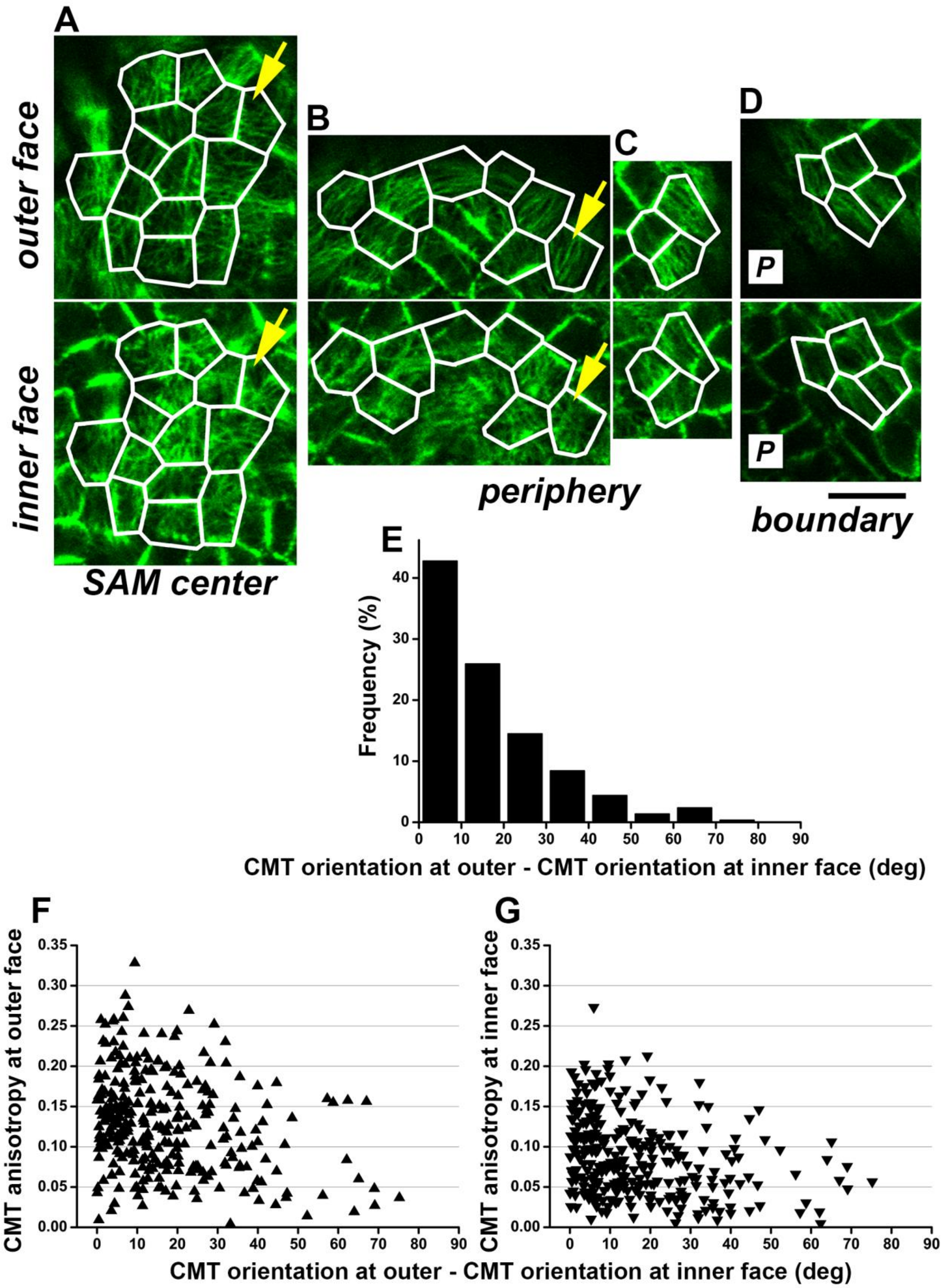

Supplementary Figure S4. CMT organization at the outer and inner periclinal faces of L1 layer. (A), (B), (C), (D) CMTs at the outer face (upper panel), and inner face (lower panel).

Cells from (A) SAM center, (B) and (C) periphery, (D) boundary between SAM apical dome and primordium (P) are shown. The same cells are outlined in white at upper and lower panels. Yellow arrows indicate the cells, at which CMT organization is different at outer and inner faces. Bar = 10 µm. (E) Histograms of the difference between mean CMT orientation at the outer and inner cell faces (absolute vales). (F) and (G) The difference between mean CMT orientation at the outer and inner cell faces plotted against anisotropy of CMT array at the outer (F) and (F) inner faces.

Supplementary Movie 1. CMT organization across the SAM layers. Note the supracellular alignment of CMTs in the boundary domains on both outer and inner cell faces of the L1 cells.

Supplementary Results 2. Relation between CMT orientation and maximal growth direction for cells in the selected SAM domains.

The selected domains are similar to those selected in Figure 3. At the domain CZ Gaussian curvature was low but positive (Supplementary Fig. S5, A and B). The dispersion of both CMT orientation and maximal growth direction among cells was relatively high, and CMTs were randomly oriented with respect to the growth direction (Supplementary Fig. S5, C and D).

The PZ' domain is located between the bulging primordium (3) and CZ domain (Supplementary Fig. S5, A to C). CMT orientation tended to be latitudinal, while the growth is meridional, thus there was a tendency to orient CMT perpendicular to maximal growth direction (Supplementary Fig. S5E). Both domains PZ'' and PZ''' were adjacent or partially localized at the position where initial bulges (1) and (2) have formed. The PZ'' represents the "earlier" stage, while the PZ''' – the "later". Consequently, at this domains Gaussian curvature increased at the second time point in comparison with the first one. At PZ'' domain, maximal growth direction was mainly in the latitudinal direction, but still the dispersion of CMT orientations was relatively high and the mean CMT orientation was changing between two time points. CMTs were temporarily either parallel or perpendicular to the growth direction. At PZ''' domain growth was mainly in the meridional direction and CMTs were mainly of latitudinal orientation. Thus, CMTs were oriented rather perpendicularly to maximal growth direction.

At the domain B', where boundary formation took place (note negative Gaussian curvature at the second time point in comparison with the first [Supplementary Fig. S5, A to C]), both CMT orientation and maximal growth direction were in the latitudinal or nearly latitudinal direction (Supplementary Fig. S5F). Thus, CMTs were mainly parallel to maximal growth direction.

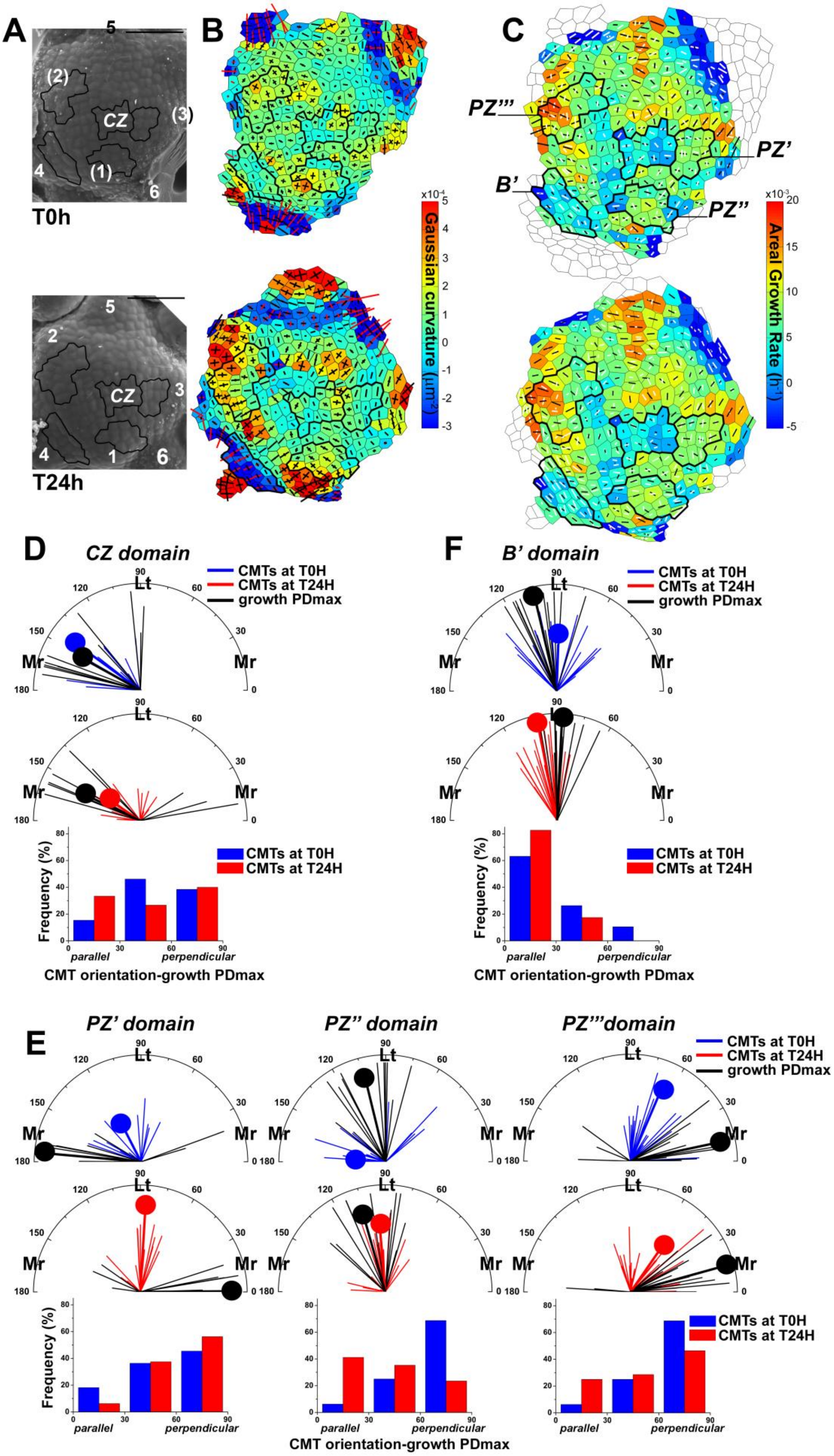
A
B
C
T0h
T24h
CZ
PZ'''
PZ'
PZ''
B'
Gaussian curvature (µm⁻²)
Areal Growth Rate (h⁻¹)
D
CZ domain
F
B' domain
CMTs at T0H
CMTs at T24H
growth PDmax
Lt
Mr
Frequency (%)
parallel
perpendicular
CMT orientation-growth PDmax
E
PZ' domain
PZ'' domain
PZ'''domain

Supplementary Figure S5. Relation between CMT orientation and maximal growth direction for cells in the selected SAM domains. (A) Scanning electron micrograph of the representative SAM at the first (T0 h, upper panel) and the second (T24 h, lower panel) time points. Primordia are numbered starting from the youngest (numbers of incipient primordia are in brackets). The selected domains are outlined in black. Bar = 50 µm. (B) Corresponding curvature maps at the first and the second time points. Gaussian curvature is represented in the color scale; curvatures in principal directions - as cross arms (for positive values in black, for negative in red). (C) Growth rate maps and overlaid CMTs. Areal growth rate is represented in the color scale, maximal growth direction (growth PDmax) – as a black line segment, the mean CMT – as a white. These parameters were plotted onto the cell outlines at the first (T0 h, upper panel) and the second (T24 h, lower panel) time points. (D), (E), (F) Polar plots of CMT orientation and the direction of growth PDmax for the first (T0 h, upper panels) and the second (T24 h, middle panels) time points at CZ (D), PZ (E), and boundary (F) domains. Thin line segments represent CMT orientation (blue for T0 h, red for T24 h) or growth PDmax (black) in individual cells, the segment length is proportional to CMT or growth anisotropy. Thick line segments (tipped with a dot) represent circular mean CMT orientation or mean growth PDmax (weighted by the anisotropy), its length is the measure of a dispersion of data: the longer the segment, the more data are concentrated around the mean orientation/direction. (Mr) refers to meridional CMT orientation or growth PDmax (0^0 - 30^0; 150^0 - 180^0 with respect to meridional SAM direction); (Lt) – to latitudinal (60^0-120^0). Histograms of the difference between CMT orientation and the growth PDmax for the first (T0 h, blue bars) and for the second (T24 h, red bars) time points (lower panels) at CZ (D), PZ (E), and boundary (F) domains. CMT orientation is regarded as parallel to the growth PDmax for the difference 0^0-30^0, and perpendicular for 60^0-90^0. Cell numbers are: n_{CZ}=13 (T0 h) and 15 (T24 h); $n_{PZ'}$=11 (T0 h) and 16 (T24 h), $n_{PZ''}$=16 (T0 h) and 17 (T24 h), $n_{PZ'''}$=20 (T0 h) and 28 (T24 h); $n_{B'}$=19 (T0 h) and 23 (T24 h).

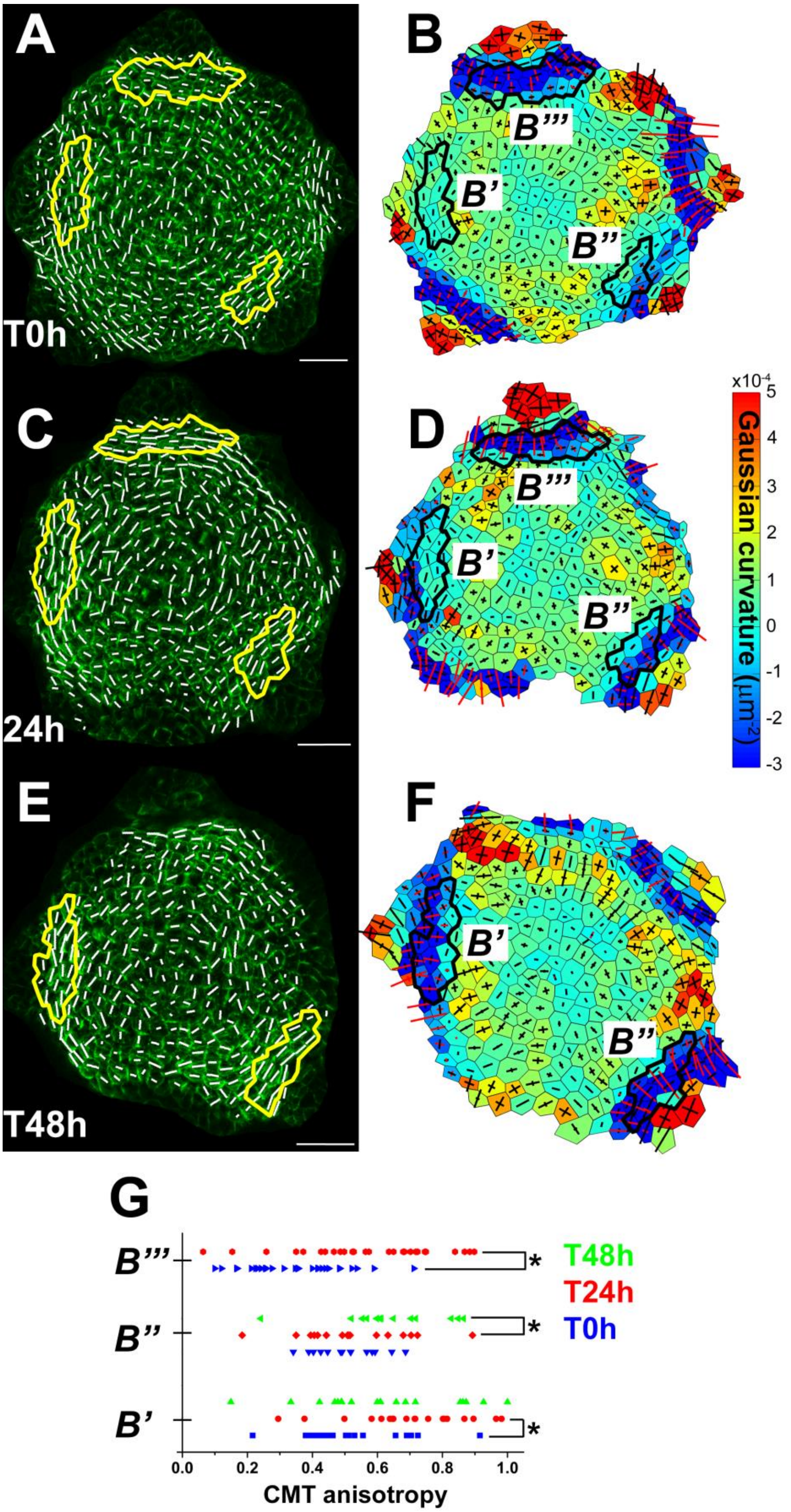
A
B
B'''
B'
B''
T0h
C
D
24h
E
F
T48h
x10-4
Gaussian curvature (µm-2)
5
4
3
2
1
0
-1
-2
-3
G
T48h
T24h
T0h
0.0
0.2
0.4
0.6
0.8
1.0
CMT anisotropy
*

Supplementary Figure S6. Changes of CMT orientation and anisotropy of CMT array during boundary formation. The boundary domains (outlined): B’ (the “youngest”), B’’, and B’’’ (the “oldest”), were identified based on the curvature and supracellular CMT pattern at the T24 h or T48 h point (C and D, E and F). The same domains were recognized at T0 h and T24 h. (A), (C), (E) CMT projections at the first (T0 h) (A), the second (T24 h) (C), and the third (T48 h) (E) time points. Mean CMT orientation in a cell and anisotropy of the array are visualized as a white line segment. Bar = 20 µm. (B),(D),(F) Corresponding curvature maps at the first (T0 h) (B), the second (T24 h) (D), and the third (T48 h) (F) time points. (G) Anisotropy of CMT arrays in individual cells in the outlined boundary domains at T0 h (blue), T24 h (red), and T48 h (green). Asterisks indicate the significant difference between the distributions (Kolmogorov-Smirnov test at p=0.05). Cell numbers are: $n_{B'}$=19, 22, and 19; $n_{B''}$=14, 16, and 13; $n_{B'''}$=28 and 27, at T0 h, T24 h, and T48 h, respectively.

Supplementary References

Crowell EF, Timpano H, Desprez T, Franssen-Verheijen T, A-M Emons, Rolland A, Höfte H, Vernhettes S. 2011. Differental regulation of cellulose orientation at the inner and outer face of epidermal cells in the *Arabidopsis* hypocotyl. *The Plant Cell* **23,** 2592-2605.

Fujita M, Himmelspach R, Hocart CH, Williamson RE, Mansfield SD, Wasteneys GO. 2011. Cortical microtubules optimize cell-wall crystallinity to drive unidirectional growth in *Arabidopsis*. *The Plant Journal* **66,** 915–928.
